# Potential repurposing of oncology drugs for the treatment of Alzheimer's disease

**DOI:** 10.1186/1741-7015-11-82

**Published:** 2013-03-26

**Authors:** Wataru Araki

**Affiliations:** 1 Department of Demyelinating Disease and Aging, National Institute of Neuroscience, National Center of Neurology and Psychiatry, Kodaira, Tokyo 187-8502, Japan

**Keywords:** Alzheimer's disease, amyloid β-peptide, disease-modifying drugs, drug repositioning

## Abstract

Alzheimer's disease (AD) is the most common form of neurodegenerative dementia, affecting about 30 million people worldwide. Despite recent advances in understanding its molecular pathology, no mechanism-based drugs are currently available that can halt the progression of AD. Because amyloid-β-peptide (Aβ), a primary component of senile plaques, is thought to be a central pathogenic culprit, several disease-modifying therapies are being developed, including inhibitors of Aβ-producing proteases and immunotherapies with anti-Aβ antibodies. Drug repositioning or repurposing is regarded as a complementary and reasonable approach to identify new drug candidates for AD. This commentary will discuss the clinical relevance of an attractive candidate compound reported in a recent paper by Hayes *et al*. (*BMC Medicine *2013) as well as perspectives regarding the possible repositioning of oncology drugs for the treatment of AD.

See related research article here http://www.biomedcentral.com/1741-7015/11/81

## Introduction

Alzheimer's disease (AD) is a major type of dementia that afflicts about 30 million people worldwide [1]. Pathologically, this disease is characterized by the presence of senile plaques, composed primarily of amyloid β-peptide (Aβ), and neurofibrillary tangles, composed mainly of phosphorylated tau protein, as well as loss of synapses and neurons [2]. The few drugs currently approved for clinical use, such as donepezil, provide only symptomatic and short-term benefits [3]. Because the accumulation of Aβ, particularly the oligomeric species, appears to play a primary pathogenic role in AD [4,5], it is a primary target for disease-modifying therapeutics of AD. Aβ is generated by serial cleavages of its precursor, amyloid precursor protein (APP), by β-secretase (or BACE1) and γ-secretase [6]. Thus, inhibitors or modulators of these proteases are being developed. Other possible interventions include Aβ immunotherapy to promote Aβ clearance and inhibition of Aβ oligomerization [3,7]. No such drugs have yet been proven clinically effective, despite the urgent need to find new treatments for AD. It, therefore, seems an attractive strategy to assess the anti-Aβ effects of drugs approved for the treatment of other diseases. Such a "drug repositioning" or "repurposing" approach is thought to have several advantages, including reduced time and costs necessary for clinical trials [8,9]. A paper by Hayes *et al*. published recently in *BMC Medicine *[10] describes an interesting oncology drug that may potentially be used as a disease-modifying drug in patients with AD.

## A new candidate drug for the treatment of AD

There have been several observations from multiple studies that suggest an inverse relationship between cancer and AD; cancer patients have been shown to have a lower risk of developing AD, and similarly, those diagnosed with AD seem to have a lower risk of developing cancer [11,12]. Therefore, it seemed plausible that anticancer drugs may exert favorable effects on AD. By screening approximately 90 FDA-approved oncology drugs, a group led by Madepalli Lakshmana found that BCNU (1,3 bis(2-chloroethyl)-1-nitrosourea or carmustine), an alkylating agent currently used to treat patients with brain tumors, such as malignant gliomas, has potent activity in reducing Aβ production by cultured cells overexpressing APP [10]. Subsequent analysis of the mechanisms by which BCNU reduces Aβ production found that BCNU increases the secretion of APPα, a protein resulting from alternative α-cleavage of APP within the Aβ region, decreases the levels of C-terminal fragments of APP and increases the expression of immature APP on the cell surface. BCNU did not directly affect the enzymatic activities of β-, γ- and α-secretases. Accordingly, BCNU appears to reduce Aβ by altering the trafficking and processing of APP without directly affecting secretase activities. In addition, BCNU was found to increase transforming growth factor (TGF)-β1 levels in cell media and cell extracts, an intriguing observation in view of the involvement of the TGF-β1 pathway in AD [13].

Following these cell-based experiments, the authors performed *in vivo *experiments to determine whether BCNU could reduce Aβ production in a transgenic mouse model, in which Aβ plaques appear as early as six months of age. Intraperitoneal injection of 0.5 mg/kg BCNU, a non-toxic dose, for 60 days, from four to six months of age, resulted in the marked reduction of Aβ plaque burden in the brain. Furthermore, BCNU treatment decreased levels of Aβ and APP C-terminal fragments and increased levels of secreted APPα in mouse brains, recapitulating the changes observed in cell cultures. Moreover, BCNU treatment reduced the number of Iba1-positive microglia, indicating that this agent suppresses microglial activation in the mouse brain. This effect may be related to the TGF-β1 pathway, since TGF-β1 plays a constitutive role in the suppression of inflammation [14]. A modest increase in astroglial TGF-β1 production in APP transgenic mice has been shown to result in a significant reduction of Aβ [15]. Moreover, a specific impairment of the TGF-β1 signaling pathway has been demonstrated in the AD brain, and TGF-β1 has been found to exert neuroprotective effects against various insults, including Aβ toxicity [13,14]. Thus, BCNU treatment may reduce Aβ production through combined effects on APP trafficking and processing and on the TGF-β1 pathway.

## Oncology drugs have potential utility for AD

The study by Hayes *et al*. identified BCNU as a potential anti-Aβ drug [10]. However, several questions need to be investigated, including whether chronic treatment with BCNU can prevent cognitive impairment in the mouse model and whether BCNU up-regulates TGF-β1 *in vivo*. Because BCNU is metabolized very rapidly in the brain, the authors suggested that its anti-amyloidogenic effects may result from the action of one of its metabolites [10]; however, such a compound remains to be identified. Safety is a major issue to be considered. For the treatment of brain tumors, patients are implanted with BCNU wafers to avoid systemic side effects. Although BCNU exhibited a robust Aβ-reducing effect *in vivo *at non-toxic concentrations, the safety of long-term use has not yet been established. If a metabolite of BCNU with a potent anti-Aβ action is identified, a safer compound with less toxicity may be developed.

Potential AD therapy with existing oncology drugs appears to be a promising strategy. For instance, treatment with bexarotene, an agonist of retinoid X receptors (RXRs) that is used to treat patients with T cell lymphoma, was recently found to lead to pathological and behavioral improvements in transgenic mouse models of AD [16]. Bexarotene stimulates the expression of apolipoprotein E (ApoE) and its lipid transporters ABCA1 and ABCG1, and facilitates Aβ clearance in an ApoE dependent manner. This agent also appears to promote microglial phagocytosis. Thus, RXR agonists may be of therapeutic utility in the treatment of AD. In addition, drugs that activate retinoic acid receptors (RARs), such as acitretin and tamibarotene, which are used to treat psoriasis and acute promyelocytic leukemia, respectively, have been reported to have beneficial effects on APP processing by enhancing non-amyloidogenic α-secretase processing of APP, probably through the induction of α-secretase (or ADAM10) expression [17,18]. Because the administration of RAR agonists to APP transgenic mice decreases Aβ levels in the brain [17,18], they are promising candidate drugs for the treatment of AD. Phase II clinical trials are underway to validate their clinical efficacy. Other oncology drugs with potential utility for AD include imatinib and paclitaxel, but both have the drawbacks of poor central nervous system penetration [9].

## Conclusions

BCNU is a unique new addition to the list of candidate oncology drugs with potential clinical efficacy in AD. More preclinical work, including clarification of its mechanisms of action, is essential before BCNU proceeds to clinical trials. Needless to say, various classes of drugs other than those used for chemotherapy are regarded as potential candidate drugs for AD [8,9]. Future rigorous efforts to develop disease-modifying drugs through drug repositioning or repurposing may lead to the emergence of successful therapies for AD.

## Abbreviations

Aβ: amyloid β-peptide; AD: Alzheimer's disease; ApoE: apolipoprotein E; APP: amyloid precursor protein; BCNU: 1,3 bis(2-chloroethyl)-1-nitrosourea or carmustine; RARs: retinoic acid receptors; RXRs: retinoid X receptors; TGF: transforming growth factor

## Competing interests

The author declares that they have no competing interests.

## Author's information

WA is a neurologist and is a section chief at the Department of Demyelinating Disease and Aging, National Institute of Neuroscience, National Center of Neurology and Psychiatry (Japan).

## Pre-publication history

The pre-publication history for this paper can be accessed here:

http://www.biomedcentral.com/1741-7015/11/82/prepub
